# Realistic microstructure evolution of complex Ta-Nb-Hf-Zr high-entropy alloys by simulation techniques

**DOI:** 10.1038/s41598-019-52170-0

**Published:** 2019-11-08

**Authors:** Shashank Mishra, Soumyadipta Maiti, Balarama Sridhar Dwadasi, Beena Rai

**Affiliations:** TCS Research, Tata Research Development and Design Center, 54-B Hadapsar Industrial Estate, Hadapsar, Pune, 411013 Maharashtra India

**Keywords:** Atomistic models, Metals and alloys

## Abstract

Over last 15 years high-entropy alloys (HEAs) and complex concentrated alloys (CCAs) have gained much appreciation for their numerous superior properties. In this paper we have shown a novel simulation methodology to realistically predict the nanometer level local structural features of complex Ta_0.25_Nb_0.25_Hf_0.25_Zr_0.25_ HEA. This involves prediction of the morphology of the short-range clustering (SRCs), their quantitative atomic composition at the nano level and the thermodynamic aspects. An alloy structure model containing 11664 atoms was created and this was subjected to structure evolution at 1800 °C. The structure evolution technique is based on a combined hybrid Monte Carlo and molecular dynamics (MC/MD) approach. The simulated results from this work are further validated against experiments and material characterizations reported in literature and done by high-resolution transmission electron micrograph (HRTEM) for the nano-level microstructure, atom probe tomography (APT) for the local chemical compositions and X-ray diffraction at synchrotron sources for the local lattice relaxation effects. This work qualitatively and quantitatively reproduces the materials characterization results reasonably well from the developed simulation methodologies. The structure evolution methods as described in this work are based on independent computer simulations and does not involve any manual intervention for input based on experiments on evolving SRCs. This work shows the potential of utilizing MC/MD based computational methods to reduce the number of costly experimental characterizations and accelerate the pace for materials development.

## Introduction

The concept of high-entropy alloys (HEAs) was materialized by Yeh *et al*. and Cantor *et al*. around 15 years ago^1,2^. In recent years they have gained popularity for their simplistic bcc or fcc type average structure and a wide range of exciting functional and engineering properties^1–4^. HEAs are loosely defined as alloys containing 4 or more constituent elements with body-centred cubic (bcc) or face-centred cubic (fcc) type average structure in equiatomic or near-equiatomic ratio in various literature^1–4^. Some of the very interesting properties that have been observed in HEAs are high strength and ductility at both ambient and high temperatures, which can be even better than many engineering materials like Ni-based Superalloys, Hastelloy and Stellite; hardness and wear resistance comparable to bearing and tool steels^1,4–6^. Also other functional properties found in HEAs offer their potential use in diffusion barriers in microelectronics^7^, binder material for cutting tools^8^, as cryogenic and radiation tolerant materials etc^9,10^. Researchers often associate the words concentrated solid solution alloys (CSSAs), multiprincipal element alloy (MPEA) and complex concentrated alloys (CCAs) with HEAs, but the HEAs have single-phase average structures which should not be confused with other multi-phase alloys^11,12^. Due to their high configurational entropy, given as *Rln(n)*, where *R* is the universal gas constant, *n* being the number of constituent elements; the entropic contribution to the Gibbs free energy of HEAs suppresses the formation of intermetallics and hence stabilises the solid-solution alloy^1–4,11^. Many of the promising superior properties as observed in the HEAs have been attributed to the distorted bulk lattice framework structure and low stacking fault energy^9,12,13^. HEAs can be easily synthesized unlike conventional single principal element alloys, which usually have problems of complex microstructures resulting in brittleness, drop in strengthening at elevated temperature and long-term use in high temperature, precipitation of other intermetallic phases etc^1,3,5^.

The common HEAs reported in the literature are mostly based on elements like Al, Co, Cr, Cu, Fe, Ni, which have shown comparable or better properties than that of steels, Inconel etc^5,14^. To achieve higher strengths at high-temperature regime above 1100 °C-1200 °C, the use of refractory metals in HEAs was implemented by Senkov *et al*. in 2010^15^. These refractory HEAs like MoNbTaW, TaNbHfZrTi etc are especially important for the development of high-temperature sustaining materials for aerospace applications^15–17^. Refractory metals and refractory HEAs have also shown their higher strengthening features at ambient temperatures, potential for use in electrical resistors, medical implants etc^15–18^. In the literature some refractory HEAs with single phase bcc average structures have been reported in the as-cast or homogenized conditions^15–19^. But, there are very few literature available for the long term phase stability at high-temperature, long-range or short-range ordering/ clustering (SRO/ SRC) on the nano-scale which can affect their applicability as high-temperature structural materials. However, in recent years a refractory HEA Ta_0.25_Nb_0.25_Hf_0.25_Zr_0.25_ system has been experimentally studied for their local nano-structure evolution, SRCs and the effect of high-temperature annealing on mechanical properties by Maiti *et al.*^19^. For this TaNbHfZr alloy, it was observed that with increasing annealing time there was formation of SRCs with evolving morphologies, which dictated the mechanical properties of this single-phase alloy. The average structure type, lattice parameters and local real structure, local chemical compositions with respect to high-temperature annealing has been well characterized. The effect of long term annealing heat treatment on the mechanical properties such as hardness, strength and ductility has also been experimentally measured. However, for all the experimental characterizations, lots of high-end expensive and time consuming, cumbersome and labour intensive techniques and equipment such as high-resolution transmission electron microscopy (HRTEM), atom-probe tomography (APT), synchrotron source single-crystal X-ray diffraction, neutron powder diffraction etc were used^19^.

With the advent of computer simulations techniques such as molecular dynamics (MD) simulations, Monte Carlo (MC) simulations, density functional theory (DFT) based electronic structure calculations, researchers nowadays are trying to obtain the nanometer level real structure and the bulk lattice structures of HEAs using simulation techniques^11,20–22^. These are mainly used to simulate growth of HEA thin films by MD^21^, average structure stability and non-equilibrium mechanics by DFT and MD^11,20^, diffusivity in amorphous liquid HEAs by ab-initio MD (AIMD)^22^ etc. These DFT and MD simulation methods are severely limited by the timescale of picosecond and nanosecond level; and lengthscales by few hundreds and few million atoms, respectively. So, much about the nanostructure morphology, SRO/ SRC in the bulk nanostructure and related properties cannot be investigated by using simple DFT and MD. To overcome this difficulty of dealing with tiny lengthscale and timescales involved in DFT and MD techniques, some accelerated dynamics techniques were developed in combination with Monte Carlo based MD (MC/MD) techniques^23^. It was generally found that the different types of hybrid sequential or mixed MC/MD techniques can accelerate the dynamics by a factor of 10^7^, which would transform the dynamics timescale of MD simulation from the nanosecond/ microsecond to the more practical and realistic seconds timescale^23^. Inspired by these MC/MD methodologies, ab-initio based MC/MD simulations were tried on HEAs for the theoretical investigation of SRO/ SRC effects, prediction of equilibrium intermetallic phases and limited information about lattice distortion effects by Widom *et al*. in recent years^24,25^. Refractory HEAs like MoNbTaW, NbTiVZr and CrMoNbV systems were investigated in literature by MC/MD techniques, but the simulation system had just a handful of 128–256 atoms, spanning for around 1–2 nm in length-scale^24,25^. This did not provide ample scope of doing proper structural and morphological studies even at the nanometer scale because the DFT based energy calculations took significant amount of computation time at each step of MC/MD. There is a lack of literature in which classical MD based technique has been used in MC/MD based approach for HEAs, by which many thousands of atoms with tens of nanometer long system sizes could be simulated for their local real structure evolution.

In literature, an EAM potential for TaNbHfZr HEA was developed and using this, local structural relaxation at the modeled SRCs, changes in local enthalpy and its relation to the extra mechanical strengthening was studied Maiti *et al*.^19^. But in that case a presumed model simulation system was initially manually constructed based on the information about SRCs obtained from detailed high-end experimental characterizations and interpretations from HRTEM and APT. There is a lack of literature in the fields of HEAs or CCAs where the local lattice structure was evolved by independent computer simulation with no experimental input; structural relaxation, SRO/ SRC and thermodynamic properties determined to explain the real behaviour of the alloy under certain heat-treatment processing conditions etc. In this work, we have computationally studied equimolar TaNbHfZr HEA for its nanostructure morphology evolution for high-temperature annealing, evolution of SRO/SRC, local lattice relaxations, which closely validates these structural characteristics obtained from the abovementioned high-end experimentations like HRTEM, APT and synchrotron XRD^19^. In this work the TaNbHfZr system had 11664 atoms with 18 × 18 × 18 bcc supercell structure, which including its periodic neighbours can span a cubic length of around 19 nm, and was found to be sufficient for the simulation of nanostructure and SRC evolution with high-temperature annealing treatment at 1800 °C. MC/MD technique with EAM type MD potentials were utilized in this work.

## Results

### Nanostructure morphology

**Figure 1:**
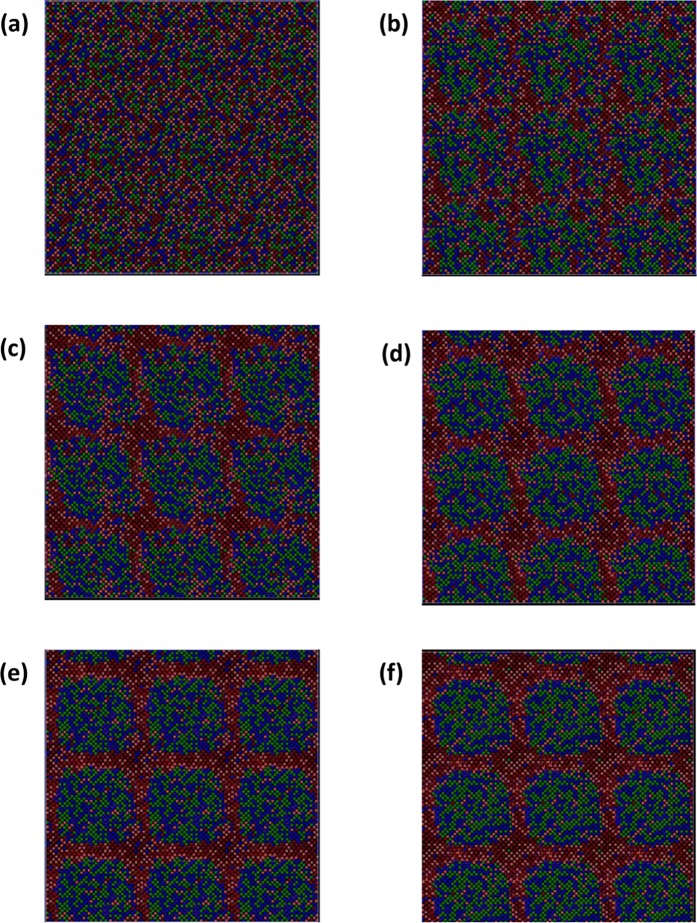
MC/MD evolved structures with different degree of attempted MC swaps per atom: (**a**) 0 swaps, (**b**) 10 swaps, (**c**) 15 swaps, **(d**) 25 swaps, (**e**) 35 swaps and (**f**) with 50 swaps, respectively. Ta, Nb, Hf and Zr atoms are depicted with green, blue, orange and maroon colours, respectively.

At first an equimolar TaNbHfZr HEA initial random structure was created with a bcc lattice framework. The atomic sites of the created 18 × 18 × 18 supercell structure was randomly assigned the 4 different atom types. The lattice parameter (LP) at 1800 °C was found to be 3.54 Å and this LP was used for all the MC/MD evolved structures. The details of the simulation procedures are given in the Methods section. The system had 11664 atoms and a total number of 583200 MC trials were performed, giving rise to up to 50 attempted MC swaps per atom. In Fig. 1, the evolved nanostructures are displayed for the different stages of structural evolution. The simulation box along with its periodic neighbours is presented. Figure 1a shows the initial random substitutional alloy structure and the Fig. 1b–f shows the alloy structures evolved after 10, 15, 25, 35 and 50 attempted MC swaps per atom (henceforth called MC swaps), respectively. At a first glance, it can be noticed that with increasing number of attempted swaps, the maroon and orange (Zr and Hf) atoms have a tendency of clustering together. After around 15 MC swaps, the Zr and Hf rich atomic co-clusters of SRCs become much prominent and it appears that they appear perpendicular to the <1 0 0> crystallographic directions. At around 25 MC swaps it was observed that the SRCs grow larger perpendicular to the <1 0 0> directions and gradually become interconnected with each other. As shown in Fig. 1c, as the structure evolves further, the smaller Zr and Hf-rich SRCs tend to fuse into a wide band like SRC, which grows independently along the X, Y and Z planes and ultimately touches each other at nodes. The Ta and Nb atoms also cluster together in adjacent regions, which gets isolated and forms a cuboid-like region later on with more MC swaps. Near the node the SRCs look like forming a cross-type pattern, which was also experimentally found in the system during annealing treatment at 1800 °C as characterized by HRTEM and APT^19^.

**Figure 2:**
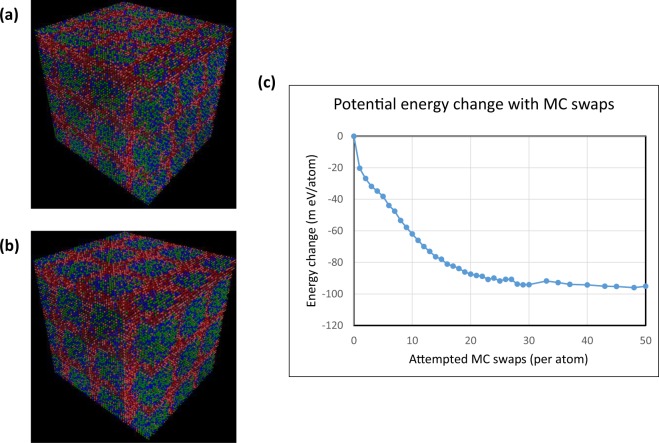
Perspective views of MC/MD evolved structures and the corresponding change in enthalpy: (**a**,**b**) are evolved after 15 and 50 atomic MC swaps, respectively. **(c**) shows gradual change of potential energy from the initial structure to 50 MC swap evolved structure. Ta, Nb, Hf and Zr atoms are depicted with green, blue, orange and maroon colours, respectively.

Figures 2a,b shows the MC/MD evolved structure from a perspective view for systems evolved for 15 and 50 MC swaps. It can be viewed that the SRCs rich is Zr and Hf have evolved perpendicular to all the three principal crystallographic <1 0 0> type directions. These interconnected SRCs have created isolated Ta and Nb rich regions which look like nano-scale cuboid-like structures. As the MC/MD swaps proceed from 15 to 50 swaps, it is found that the nature and morphology of the SRCs present in the system largely remains similar. The only significant difference that could be found here is in the coarsening of the nodal regions of SRCs where 2–3 SRCs meet each other. The change of enthalpy of the evolving structure with respect to the initial random structure during MC/MD moves is shown in Fig. 2c. The energy of the evolving structures reduces rapidly by 60 m eV/atom within first 10 MC swaps and by 80 m eV/atom by 15 MC swaps. After this, the rate of change of energy of the structure becomes small, only by 15 m eV/atom more until 50 MC swaps. This is also in accordance with the relative stability of the morphology of the structure after 15 MC swaps shown in Fig. 2a,b.

**Figure 3:**
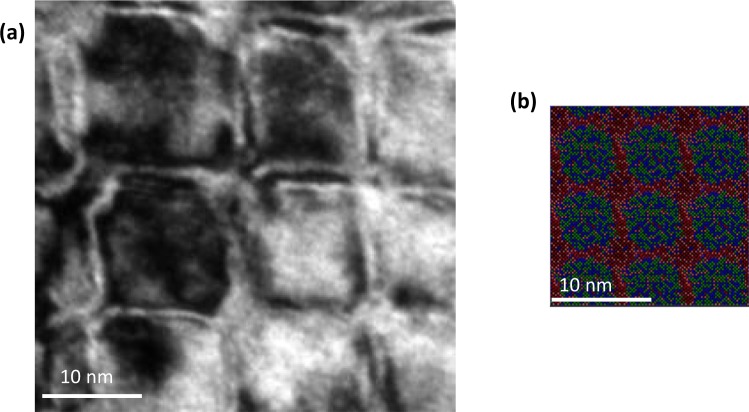
Comparison of real HRTEM and simulated microstructure: (**a**) HRTEM bright field image of TaNbHfZr HEA annealed at 1800 °C for 4 days oriented along [1 0 0] zone axis and principal lattice directions. (**b**) corresponding scaled simulated atomic structure evolved after 25 MC swaps. Ta, Nb, Hf and Zr atoms are depicted with green, blue, orange and maroon colours, respectively. (**a**) adapted from ref.^19^ with permission from Elsevier.

**Figure 4:**
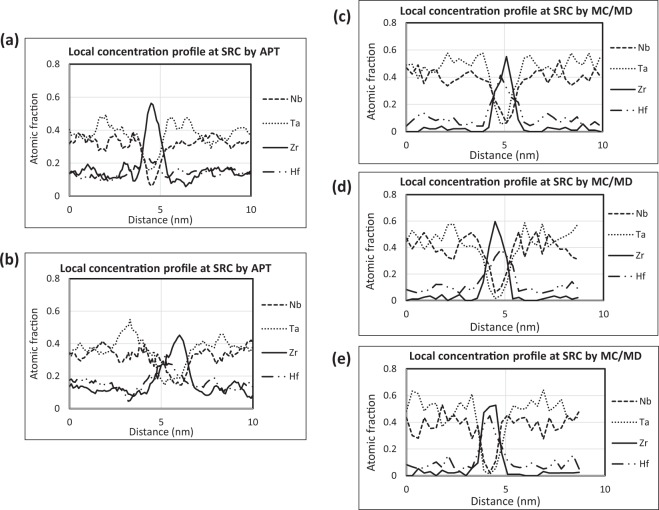
Local composition scan from APT and MC/MD simulated structure: (**a**) and (**b**) local composition scans by APT perpendicular to SRCs present in HEA annealed for 4 days from 2 different areas. (**c**–**e**) are composition scans along X, Y and Z directions of 25 MC swap evolved structure, respectively.

The nature of local nanoscale structure evolution and SRCs, as predicted by MC/MD method, can be validated by the observed HRTEM and the APT atom-map reconstructions in literature^19^. In Fig. 3 bright-field HRTEM micrograph and the related MC/MD evolved structures are provided with same <1 0 0> crystallographic orientation for comparison purpose. The HRTEM is for the HEA annealed for 4 days and the simulation evolved structure is taken after 25 MC swaps as at this point the annealed and simulated nanostructures appear very similar in their morphology. In ref.^19^. about this HEA, it could be found from HETEM and APT based experiments that the independently evolving isolated SRCs gradually grow in {1 0 0} habit planes and touch each other and make the structure look like total grid pattern after 4 days of annealing. This is analogous to the sequence of simulated structures forming interconnected cross-like grid pattern sequence by reaching 25 MC swaps. In Fig. 3a the real structure SRCs have become fully interconnected with each other to form repetitive domain-like morphologies, dividing the whole space into tiny cuboid-like domains as reproduced in simulations in Fig. 3b. However, since the HRTEM does not exclusively provide information about the local nanoscale chemical composition qualitatively or quantitatively, the MC/MD evolved structures are also compared with APT composition scans in Fig. 4.

### Local chemical composition

The nanostructure evolution by high-temperature annealing treatment of the HEA, as analysed by MC/MD simulations and comparisons with HRTEM and APT atom-maps^19^ provide a qualitative agreement of the nature of SRCs evolved. Here the local chemical composition of each element of the alloy across the width of the SRCs and the surrounding regions, as obtained from APT and MC/MD simulations are analysed and compared in Fig. 4. For the APT based composition profile of the 4-days annealed sample, a cylindrical region was chosen which goes through the SRCs and whose axis is almost perpendicular to the SRC forming plane. Then the atoms falling inside the cylinder are binned at a distance of 1 Å and plotted in Fig. 4a,b, from two different parts of the annealed alloy. Figure 4c,e are the local atomic concentration profiles of the MC/MD simulated system, taken along the three principal crystallographic directions. The simulated system with 25 MC swaps was chosen for comparison with the 4-days annealed alloy because of the close similarity of their nanostructure morphology (Fig. 3). The atomic composition scan from the APT reconstruction shows the strong prevalence of Zr at the SRCs and a weaker co-clustering effect with Hf. The 1-d composition profile of Zr perpendicular to the selected SRC in the APT analysis shows a Gaussian type composition variation. The width of the cluster from APT is around 1–1.5 nm as measured at the FWHM level of the concentration distribution^19^. The concentration profiles of the elements from the MC/MD simulation was also found to be very similar to as found in the APT study with the SRCs being rich in Zr with just a lesser degree of co-clustering with Hf. The width of the SRC obtained from MC/MD simulation is close to 1–1.2 nm as observed for the APT and the peak composition of Zr is also similar for both MC/MD and APT. The Ta and Nb concentrations are depleted at the Zr-Hf rich SRCs evidenced from composition scan from both APT and MC/MD simulations. As the SRCs are depleted in Ta and Nb, their concentrations increased at the adjacent regions, as evident from both experimental and simulation results.

### Local structural relaxation

**Figure 5:**
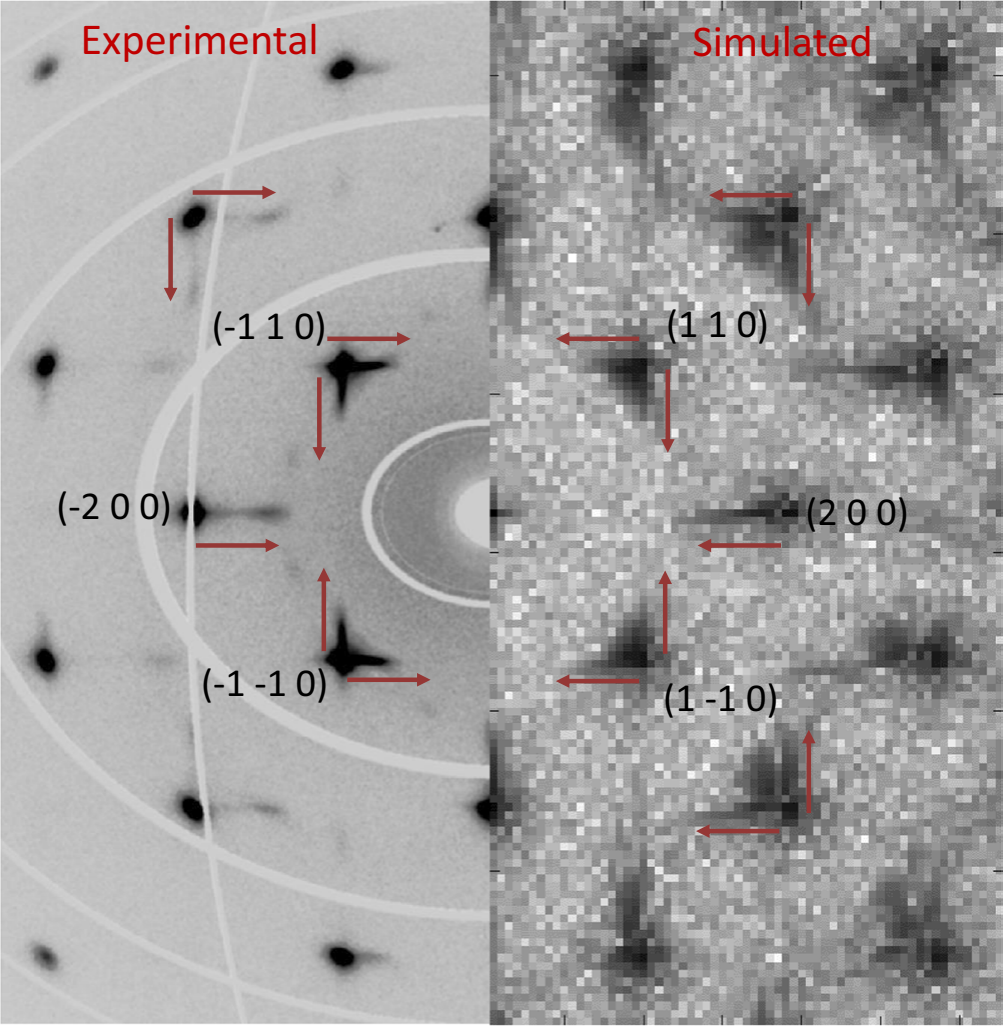
Comparison between the single-crystal XRD obtained *hk0* reciprocal lattice layer of 4-days annealed sample with the 25 MC swap evolved one: The diffuse scattering streaks emerging asymmetrically from the Bragg reflection spots are shown with little arrows. The experimental left side portion of the reciprocal lattice layer is adapted from ref.^19^ with permission from Elsevier.

The local structures of alloys are experimentally characterized usually by powder or single-crystal X-ray diffraction analysis. The currently studied TaNbHfZr system has been experimentally studied in literature by various diffraction techniques to investigate into the average-structure lattice framework, atomic displacement parameters (ADPs) and diffuse scattering caused by any kind of SRO/ SRC type local structural disorder^19^. Single crystal diffractions done on the annealed samples of TaNbHfZr showed streak-like diffuse scattering emerging from the Bragg reflection spots. The diffuse scattering streaks are oriented along the main <1 0 0> crystallographic directions, indicating presence of disorder perpendicular to these directions, discussed in details by Maiti *et al*.^19^. Here the already MC/MD evolved structure was relaxed to its minimum ground-state energy at 0 K and then the corresponding simulated XRD pattern was calculated to avoid thermal diffuse scattering effects. The experimentally reconstructed *hk0* reciprocal lattice layer obtained from synchrotron X-ray diffraction measurements of the 4-days annealed HEA and simulated *hk0* reciprocal lattice layer of 25 MC swap evolved structures are compared in Fig. 5. It can be found that in both the experimental and simulated reciprocal lattice layer, the streak-like diffuse scatterings are emerging out from the Bragg reflection spots along <1 0 0> directions and the streaks are asymmetric towards lower reciprocal lattice vector. By studying all the simulated results with the experimental validations, it can be observed that the nano-scale morphology of SRCs in TaNbHfZr HEA bcc average structure and its L-shaped streak-like diffuse intensity in reciprocal lattice layers are quite similar to that observed in Guinier-Preston (GP) Zone type local ordering^26^. The prime reason for the formation of the SRCs in the {1 0 0} set of planes and local structural relaxation have been attributed to the elastic anisotropy of the matrix^27–30^, characterized by the anisotropy factor

*A* = *2C*_44_*/(C*_11_*-C*_12_), which is obtained from the second order elastic stiffness constants. In the studied HEA, the anisotropy factor as obtained from the MD simulation was found to be between 2.15–2.26, indicating a lower elastic strain energy along <1 0 0> directions. The elastic stiffness constants of the constitutive elements of the HEA and the alloy average structure as calculated from MD simulation are compared with experimental results in Table 1.

**Table 1:** Comparison of the second order elastic constants of the pure elements with that of the MD obtained values of pure elements as well as alloys.

Element/alloy	MD obtained values			Experimental values		
	C_11_(eV/Å^3^)	C_12_(eV/Å^3^)	C_44_(eV/Å^3^)	C_11_(eV/Å^3^)	C_12_(eV/Å^3^)	C_44_(eV/Å^3^)
**Elastic constant values for cubic system**						
Hafnium	0.8064	0.621	0.3878	0.818	0.643	0.281
Niobium	1.511	0.781	0.2112	1.529	0.824	0.177
Tantalum	1.515	1.011	0.4577	1.648	0.986	0.516
Zirconium	0.6946	0.5681	0.36	0.649	0.58	0.237
Initial HEA alloy	1.034	0.7467	0.3241	—	—	—
HEA after 10 MC swaps	1.007	0.7262	0.3223	—	—	—
HEA after 25 MC swaps	0.9978	0.7105	0.3088	—	—	—
HEA after 50 MC swaps	0.9936	0.7058	0.3097	—	—	—

## Discussion

In this work the effect of high-temperature long-term annealing of TaNbHfZr HEA has been successfully simulated and analysed for the closeness of validation with respect to real experimental characterizations. The methodologies used for simulating the HEA based on MC/MD simulations can investigate tens of thousands of atoms for their real nano-scale structure evolutions. On the TaNbHfZr system, a multi-cell Monte Carlo simulation was tried out recently by Niu *et al*.^31^. But since their technique involved DFT based *ab-initio* calculations, only a tiny structure containing 64 atoms could be simulated for predictive phase separation at low temperatures. Although efforts have been made to model local chemical SRO/SRC in HEAs for a few systems in the literature, there had hardly been any supportive experimental evidences provided as validations with respect to APT, X-ray diffuse scattering analysis, complementary HRTEM for simulated nanostructure study reported^19,24,25^. The earlier work on this HEA by Maiti *et al*.^19^ tried to address these aspects by manually introducing SRCs into the model atomic structure. But the local chemical composition of the evolved SRCs for their role on local structural distortions were based on experimental APT and HRTEM characterizations as a simple presumed model. In this work all the structure of SRCs are evolved automatically by computer using the MC/MD method with no experimental inputs. The nano-scale morphology of the evolved structure after 15 MC swaps closely resembled the actual nanostructure captured by HRTEM and APT atom map after 1 day and 4 days of annealing^19^. The early stages of ordering morphology by 10–15 MC swaps were also similar to the Zr clustering atom map obtained from APT after 6 hours of annealing. The evolved SRCs maintained a 3-d cubic symmetry (Fig. 2) in their evolved morphology as evidenced by HRTEM, APT and diffuse X-ray scattering studies established^19^. This indicates that the applied MC/MD technique not only captured the final state of the nano-scale morphology of SRCs, but it also captured the sequence of evolution of SRCs together with their morphology as compared with the experimental results. For a close and direct comparison of the simulated structure and nano-scale real microstructure, scaled and oriented images are presented in Fig. 3 for evolved morphologies. This was possible because of the sampling of the configurations using Metropolis type configuration acceptance criterion, which gradually decreased the energy of the system and progressed through the different stages of the structure evolution sequentially as shown in Fig. 2c. After each attempted atomic swap of the MC/MD trial, the local structure was subjected to energy minimization by conjugate gradient (CG) relaxation keeping the LP constant for 1800 °C. This allowed many surrounding atoms of the swapping atoms to move to relaxed positions simultaneously. The multiple local small atomic displacements involved in each CG energy minimization can be compared to many MC displacements achieved in one trial swap. This helps in accelerating the simulation of hybrid MC/MD runs as discussed earlier in the literature^23^. The surprising feature of this MC/MD structure evolution of the HEA is the realistic evolution of the SRCs along the {1 0 0} habit planes as experimentally observed. In many GP-zone forming alloys this kind of orientation relationships are found and attributed to the elastic anisotropy^26,28,29^. By analysing the simulated growth evolution of Cu-rich SRC type GP zones in Al alloys, it was found that the main factor contributing to the <1 0 0> orientation of SRC is the elastic anisotropy rather than the possible interfacial chemical energy between the SRC and the matrix^27^. Also for spinodal decomposition of alloys, the composition fluctuation and clustering found in {1 0 0} planes are also attributed to the elastic anisotropy, *A* > *1* of the matrix^30^. The MC/MD methodology presented in this work could successfully demonstrate planer clustering of Zr and Hf atoms along the {1 0 0} planes because the MD potential accounted for the elastic anisotropy of the average matrix structure like in Al-Cu alloys as well^27,28^. Table 1 summarizes the second order elastic constants of the pure elements and the alloy at different stages of evolution obtained by utilizing the EAM potential used in this work. It can be found that the MD obtained stiffness constants reproduced very well the experimental values for the pure elements, which were utilised as input to build up the EAM potentials^19^. The elastic anisotropy of the average structure is in the range 2.15–2.26, which indicates a lower elastic strain energy along <1 0 0> direction than other directions. This anisotropic factor promotes the Zr and Hf-rich SRCs to grow more in the {1 0 0} planes to form tiny local clusters and relax along <1 0 0> directions as observed both in simulation and experiments.

This type of MC/MD techniques have been reported to be more ergodic which helps to sample larger configuration space^23^. The other hybrid MC/MD studies done by Widom *et al*.^24,25^ in the field of HEAs involved *ab-initio* MD demanding lots of computing resource, ultimately limiting the total number of MC swaps and the NVT MD steps possible between the swaps. This could be the main reason why in the literature the hybrid MC/MD method based evolved HEAs and other high-concentration alloy structures were mainly limited to 128 atoms^24,25^. Other than the qualitative comparison of the nature of SRC morphology evolution, this work also investigated into the local compositions along the SRC and surrounding regions. The local chemical composition scan perpendicular to the SRCs from experiments and simulations match well in terms of the composition curve, values of elemental compositions and width of the SRCs (Fig. 4). Especially the values of Zr, Ta and Nb concentration at the SRCs match well between the APT composition scan and the MC/MD evolved structures. Only the clustering of Hf appears to be bit more pronounced in the MC/MD evolved SRCs than the experimental values. The composition scan in the simulated structures done along the 3 principal crystallographic directions along the X, Y and Z directions are plotted in Fig. 4c–e and it can be seen that there are marked similarity regarding the pattern of chemical composition profile both qualitatively and quantitatively. As Zr and Hf are having larger atomic volume than Ta and Nb, it is expected that it will have some effect in local static distortions due to atomic relaxations at the SRCs and the adjacent regions. This was experimentally evidenced by the presence of streak-like diffuse scattering intensities along the <1 0 0> directions in single-crystal XRD and selected area electron diffraction (SAED)^19^. Even similar streak-like diffuse scattering along <1 0 0> directions adjacent to the Bragg reflections are also observed in GP zone forming Al-Cu alloys^26^. As the Cu atoms are smaller than Al in size, the Cu-rich GP zone SRCs had local contraction, causing the diffuse streaks to be asymmetric and emerging towards higher reciprocal space dimensions. Here in the TaNbHfZr HEA, the diffuse scattering streaks appear to be emerging towards lower reciprocal lattice dimensions, opposite to the behaviour compared to Al-Cu alloys. This could be intuitive from the fact that in this HEA, the Zr and co-clustering Hf atoms have larger atomic volumes compared to the average alloys atoms, which in term causes tetragonal type lattice expansion relaxations at the SRCs unlike the lattice contractions at the GP zones in Al-Cu alloys. In literature the simulated XRD pattern from the manually introduced SRO structure had similar features of asymmetric streak-like diffuse intensities around the Bragg spots, but the extent of the stretch of the diffuse scattering was not as much as experimentally observed^19^. Also the thickness of the diffuse streaks did not match up so well compared to the experimentally reconstructed reciprocal lattice layer. In this study after 25 MC swaps, the MC/MD evolved structure was taken for diffuse scattering simulations because the morphology of the structure looked similar to that observed after 4 days of annealing in close comparison to the HRTEM in Fig. 3. The calculated diffraction intensity in the *hk*0 reciprocal lattice layer was compared to the corresponding experimental results obtained from single-crystal XRD at ambient temperature (Fig. 5). The thermal Debye-Waller factor was kept as zero and the MC/MD evolved structure was relaxed to 0 K to remove the effects of thermal diffuse scattering. The diffuse scattering intensities from the simulated reciprocal layer is only dependent on the local static atomic displacements and substitutional disorder^32^. This will help to better analyse diffuse intensities from higher order reciprocal space to be compared with experimental intensities. It can be observed that in the simulated *hk*0 layer the streak-like diffuse intensities are getting elongated asymmetrically towards lower reciprocal lattice vector to the extent comparable to the experimentally obtained *hk0* layer (shown with small arrows in Fig. 5). Also the thickness of the simulated diffuse streaks is comparable to the experimental ones. This indicates that the average structural relaxation effects from the matrix and SRCs are well reproduced even in the nano-scale simulated structures compared to the XRD based diffuse intensities originating from the whole single-crystal bulk sample of dimension 30–40 μm. The coherent domain size determined from neutron diffraction experiments of this system was 10 nm^19^, while the MC/MD simulated system size dimension was around 6.4 nm. Since the domain sizes of both real and the simulated structures were in comparable range, the simulated structure could accurately reproduce the evolution of nano-scale morphology of the SRCs, local composition scan, local structural relaxation effects when compare with the experimental results. This study of the application of hybrid MC/MD techniques for investigation into the HEAs and other high-concentration alloys can greatly reduce the need for conducting costly and cumbersome experiments like HRTEM, APT, STEM, synchrotron X-ray diffraction, neutron diffractions on single-crystals and powder samples. By reducing the number of experiments for correlative characterization studies for a system, this MC/MD technique will expedite the materials development pace by greatly reducing the development cost and time.

## Conclusion

In this work we have successfully presented a methodology to predict the nanoscale structural features in single-phase equimolar TaNbHfZr high-entropy alloy by using hybrid MC/MD technique involving 11664 atoms. The use of EAM type potentials within the scope of the developed MC/MD technique gradually minimized the internal energy of the structure at a particular annealing temperature of 1800 °C. The evolution of Zr and Hf rich short-range clustering (SRC) in the simulated structures matched with that of the experimentally determined local structures in terms of their local composition, morphology and habit planes of formation. In literature the experimentally determined features of SRCs required expensive characterization techniques like HRTEM, APT, and synchrotron X-ray diffraction and neutron diffractions etc., whereas the developed automated MC/MD methodology required no such experimental inputs. The atom maps of the simulated evolving structures also captured the sequence of structure evolutions and gradually evolved interconnectedness of the SRCs, its grid pattern and nodes. The success of the EAM potential based MC/MD technique in predicting the habit plane of formation of SRCs is attributed to the calculated elastic anisotropy of the matrix. As the larger sized Zr and Hf atoms cluster in the {1 0 0} habit plane, the local lattice experiences a tetragonal structural relaxation. This is evidenced and explained by the streak-like diffuse scattering along <1 0 0> directions observed in the simulated *hk0* reciprocal lattice layer from the atomic coordinates. This simulated *hk0* reciprocal lattice layer when compared to the experimentally reconstructed *hk0* lattice layer, indicated that the simulated nanoscale structure captured both the thermodynamic aspects and local structural disorders present in the real bulk alloy.

## Methods

Firstly, a bcc lattice framework with the atomic coordinates were created for a 18 × 18 × 18 supercell structure containing total of 11664 atoms with a LP of 3.43 Å, determined from the rule of mixture of the elemental atomic volumes. The atomic sites were randomly assigned atom types Ta, Nb, Hf and Zr with probability 0.25 by using a random number generator to create an equimolar random solid-solution structure. The exact real atomic composition in this study for Ta, Nb, Hf and Zr were 25.03%, 25.47%, 24.39%, 25.11%, respectively, which was very close to the targeted atomic composition of 25%. NPT simulations were carried out at 2073 K and 1 atm pressure for 1 ns. From this NPT simulation the lattice parameter for this system at 2073 K was evaluated to be 3.54 Å from the equilibrium simulation box dimensions. For all subsequent hybrid MC/MD simulation steps this particular equilibrium average LP obtained for 2073 K was used. For the MD simulations EAM type potential was used^33^. The energy of the system is described as

$${E}_{t}=\sum _{i}{F}_{i}(\sum _{j\ne i}{f}_{j}({r}_{ij}))+\frac{1}{2}\sum _{i,j\,(i\ne j)}{\phi }_{ij}({r}_{ij}),$$

where *E*_*t*_ is the total internal energy of the system, *F*_*i*_ is the embedding function of the atom type at position *i*, *Φ*_*ij*_ is the pairwise interaction function, *f* is the spherically symmetric electron density function, *i* and *j* are the neighbouring atoms at the vicinity of each other. The pairwise interaction of dissimilar atom types ware modelled by the widely accepted form as below^34,35^

$${\phi }^{ab}(r)=\frac{1}{2}(\frac{{f}^{b}(r)}{{f}^{a}(r)}{\phi }^{aa}(r)+\frac{{f}^{a}(r)}{{f}^{b}(r)}{\phi }^{bb}(r)),$$

where *φ*^*ab*^(*r*) is the dissimilar type interaction between atom types *a* and *b* at a distance *r*, *f*^*a*^(*r*) and *f*^*b*^(*r*) are their electron densities, *φ*^*aa*^(*r*) and *φ*^*bb*^(*r*) are the pair interaction energies of the pure element states of atom types *a* and *b*, respectively. The first publications on EAM potential was done by Daw *et al*. in 1984 in which the authors have shown that this type of potential can establish the ground state properties of materials like the lattice parameter, elastic constants, vacancy formation energy etc of single elements^33^. They also established that surface energy and atomic relaxations, H adsorption on metals can be simulated using the EAM potential. Whereas, Bangwei *et al*. have developed a next-neighbour distance based cubic spline method for EAM type pair interactions for bcc metals and alloys with inputs to the model taken from the ground state physical properties of elements^34^. The versatility of the EAM potential can be evident from the work of Zhou *et al*. where they have developed a potential for 16 different bcc, fcc and hcp type metals for a complex multi-layer nano-scale atomic vapour deposition process^35^. Based on the methods of Bangwei *et al*., this MD potential was created from elemental physical input parameters like LP, cohesive energy, unrelaxed vacancy formation energy and second order elastic constants as described and tabulated in ref.^19^ Since the lattice framework in this study is bcc, the elastic constants for Hf and Zr were taken from their high temperature bcc phase^19^. An example of the accuracy of our developed potential is evident in Table 1 where the input second order elastic stiffness of the pure elements are compared with the MD obtained values. For all the MD simulations LAMMPS package was used^36^. The LAMMPS readable format of the EAM potential data file can be found in the supplementary information section.

In the MC/MD procedure two dissimilar atom types were randomly selected and swapped with respect to their positions. Then the system was energy-minimised to a convergence level of 10^–14^ eV using conjugate-gradient (CG) method using LAMMPS for the relaxed configurations keeping the LP constant for 1800 °C. Then it was subjected to Monte-Carlo swap for which two random dissimilar type atoms were chosen in the system and their positions were interchanged. Again the system was subjected to CG minimisation relaxation with LP for 1800 °C before energy comparison with respect to the previous relaxed configuration. The acceptance of this interchange of atoms was based on probability given by the Metropolis criterion^23^:

Accept the move if *∆U* < *0* due to atomic displacements, or

Accept the move with probability *exp(−∆U/(k*_*B*_*T))* if *∆U* > *0*,

where *∆U* is the change in potential energy, *k*_*B*_ is the Boltzmann constant and *T* is the temperature.

Again two atoms were randomly picked and this process of hybrid-MC/MD was repetitively carried out until the number of attempted swaps reached 50 swaps per atoms i.e. 583200 attempted swaps. During the entire simulation the potential energy as well as the coordinates of the system were recorded at intervals.

The experimental synthesis, annealing treatment processes, characterization techniques used for this alloy have been described in literature by Maiti *et al*.^19^. From the APT reconstruction of the 4-days annealed sample, a cylindrical region was chosen which goes perpendicular to some of the SRCs. The zone along the axis of the cylinder was binned into regions of 1 Å. Then a 5 point moving average value was taken as the local composition to reduce the noise level from the raw data. For the compositions to be obtained from the MC/MD evolved structure, firstly a long rectangular parallelepiped region of size around 10 × 2 × 2 nm^3^ going through a SRC along X direction was chosen. Then the atomic coordinates inside the chosen region were binned into slices of 3 Å along the X direction for the local composition determination for each slice. Similar binning analysis were done for the Y and Z directions of the evolved structure. For the simulated X-ray diffuse scattering in the *hk0* reciprocal lattice layer of the already high-temperature evolved structures, the following formula was used^37^

$$F({\boldsymbol{h}})=\mathop{\sum }\limits_{i=1}^{N}{f}_{i}({\boldsymbol{h}}){e}^{2\pi i{\boldsymbol{h}}{{\boldsymbol{r}}}_{{\boldsymbol{i}}}}.{e}^{-\frac{B{|{\boldsymbol{h}}|}^{2}}{4}},$$

where, ***F****(****h****)* is the structure factor Fourier transform value for the XRD intensity for the reciprocal lattice vector ***h***, *f*_*i*_ is the atomic scattering factor, *B* is the Debye-Waller factor, *N* is the total number of atoms and ***r***_***i***_ is the fractional atomic coordinate for the simulated supercell structure. In this study *f*_*i*_ was taken as the atomic number of the corresponding atom type. The thermal Debye-Waller factor was taken as zero, as only the relaxed static structure at 0 K with no thermal movement of atoms was considered to avoid effects from thermal diffuse scattering and highlight the effects from static atomic displacements. The magnitude of ***F****(****h****)* was plotted in log scale for the grayscale colormap of the simulated *hk0* reciprocal lattice layer.

## Supplementary information

Supplementary Tables

## Data Availability

The datasets generated during and/or analysed during the current study are available from the corresponding author on reasonable request.
