# Supplementary material for: Realistic microstructure evolution of complex Ta-Nb-Hf-Zr high-entropy alloys by simulation techniques
Source: Sci Rep. 2019 Nov 8;9:16337. doi: 10.1038/s41598-019-52170-0 (PMC6841691; doi:10.1038/s41598-019-52170-0)
Supplement: Supplementary file 1 — Supplementary Tables [file 41598_2019_52170_MOESM1_ESM.pdf]

Realistic microstructure evolution of complex Ta-Nb-Hf-Zr high-entropy alloys by simulation techniques

Shashank Mishra[1†], Soumyadipta Maiti[1†*], Balarama Sridhar Dwadasi[1], Beena Rai[1]

[1] TCS Research, Tata Research Development and Design Center, 54-B Hadapsar Industrial Estate, Hadapsar, Pune – 411013, Maharashtra, India

* Corresponding author: Email - soumya.maiti@tcs.com , Tel- +91 7338852469

† These two authors contributed equally to this work

SUPPLEMENTARY INFORMATION

The EAM type molecular dynamics (MD) potential used for this publication is uploaded in Materials Cloud repository for the public. The file can be accessed in the following link:
https://archive.materialscloud.org/2019.0052/v1
Or in the DOI: doi.org/10.24435/materialscloud:2019.0052/v1

The EAM potential file for MD simulations is created in the LAMMPS readable format. The sequence of atoms appearing in the potential file is Hf, Nb, Ta and Zr, respectively. The energy unit is in eV and the unit for distance is in Å. A README file is also provided for the convenience of the users of the potential in Materials Cloud link given.
